# Collembola are Unlikely to Cause Human Dermatitis

**DOI:** 10.1673/031.009.0301

**Published:** 2009-03-09

**Authors:** CSH Lim, SL Lim, FT Chew, TC Ong, L Deharveng

**Affiliations:** ^1^ St George Hospital, Kogarah, Sydney, New South Wales, Australia; ^2^ Department of Biological Sciences, Faculty of Science, National University of Singapore, Singapore; ^3^ UMR5202 CNRS, Origine, Structure et Evolution de la Biodiversité, CP50, Museum National d'Histoire Naturelle, Paris, France

**Keywords:** arthropods, allergy

## Abstract

There have been several unconfirmed case reports of dermatitis caused by Collembola (springtails). We recently investigated two nurses with dermatitis suspected to be caused by *Drepanura* Schött (Collembola: Entomobryidae). IgE antibodies to Collembola proteins were not detected in sera from the nurses and skin tests with the Collembola extract and crushed whole Collembola were negative in both the nurses and volunteers. This study suggests that the springtail *Drepanura* may not cause human dermatitis and that other organisms and organic matter that are also found in the moist environment inhabited by Collembola might instead be responsible.

## Introduction

Collembola (springtails) are among the most widespread and abundant terrestrial arthropods on earth (Hopkin 1997). There have been several case reports of human dermatitis, allergy, or a crawling sensation in which Collembola have been found (Womersley 1939; Pescott 1942; Scott et al 1962; Amin 2001; Martens 2004) but it is hard to determine from the reports whether these represent true cases of human skin reactions to Collombola or delusions of parasitosis. Furthermore, other organisms such as algae, fungi, bacteria, protozoa and arthropods, and decaying organic material can often be found in the same environment favoured by Collembola such that the mere finding of Collembola does not necessarily prove a cause and effect relationship. Dasgupta and Dasgupta (1990) demonstrated Collembola feeding on the blood of newts and toads suggesting that some species of Collembola are capable of biting. However, this has not been demonstrated in humans and Pescott (1942) stated that the chewing mouthparts of most Collembola are incapable of biting humans. Hence, if Collembola does indeed cause human dermatitis, it is most likely to be due to an allergic or direct irritant effect of Collembola proteins. We recently had the opportunity to test this hypothesis in two nurses with suspected Collembola dermatitis. We present below the results of our investigation in the hope that it might contribute to a better understanding of the possible role of Collembola in human dermatitis.

## Materials and Methods

Collembola were identified in the National History Museum in Paris.

### Skin tests for reactions to Collembola extract

Collembola were crushed using a homogenizer and suspended in phosphate buffered saline (137 mM NaCl, 8.1 mM Na_2_HPO_4_, 2.7 mM KCl, 1.4 mM KH_2_PO_4_, pH 7.4) for 2 hours at 4°C. The extract was then centrifuged at 14,000 g for 10 minutes at 4°C. Supernatant was collected and the pellets discarded. Total protein concentration was then determined using the BioRad protein assay (Bio-Rad Laboratories, www.bio-rad.com) based on the Bradford method (Bradford 1976). Final preparation for skin prick testing contained 50% glycerol with total protein concentration of 0.2mg/ml. Skin prick tests were performed on the volar aspect of the forearm of the nurses and two volunteers with the Collembola (protein) extract, Bencard test solutions comprising of house dust, house dust mite, grass pollens, tree pollens, cat, dog and moulds and our own panel comprising of cockroach (American & German), shellfish (scallop, mussels & oyster), fish (tuna, sea bream, saler & mackerel) and crabs and prawns (flower crab, mud crab, tiger prawn & grey prawn). The Collembola extract and these cocktails were prepared as detailed earlier and mixed at equal volume for the skin prick tests. Histamine and saline were used as positive and negative controls, respectively. A positive test reaction was defined as a weal diameter of at least 3 mm diameter greater than the negative control. Skin crush tests were also performed by crushing whole Collembola against the skin of both the nurses and the volunteers and examined immediately and after 20 minutes. Patch tests were also performed on the backs of the nurses and volunteers with crushed Collembola in white soft paraffin and read at 48 and 96 hours. Informed consent was obtained from all subjects.

### IgE assay for antigens to Collembola in sera

Sera were collected from the nurses to test for IgE antibodies against Collembola protein. Each well in test plates were coated with total protein (10 ng) in phosphate buffered saline (PBS) overnight at 4°C. The wells were then washed three times with PBS-T (phosphate buffered saline + Triton-X). The same washing method was used throughout the assay. Blocking was carried out using PBS-T 0.1% for 30 minutes at room temperature followed by washing. 50 µ″l of patients' sera were then diluted 1:1 (v/v) with PBS and incubated in coated wells overnight at 4°C. The wells were then washed and incubated with 100 µ″l of a mouse anti-human IgE biotin conjugated (BD Pharmingen, USA) diluted with PBS-T 0.05% for 2 hours at room temperature. Wells were then washed and incubated with 1:1000 (v/v) dilution of avidin-horseradish peroxidase (BD Pharmingen, USA) in PBS-T for 30 minutes at room temperature. This was followed by washing six times before the colour substrate 3,3′,5,5′-tetramethylbenzidine (TMB) (Sigma, USA) was added and the colour intensity read at 655 nm.

## Results

**Figure 1.**
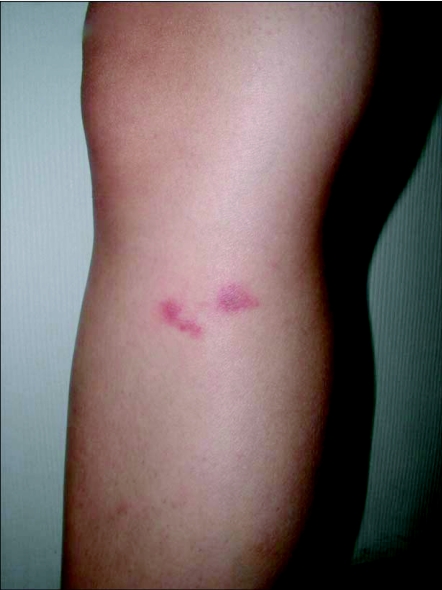
Urticarial papules on the leg of one nurse.

The nurses worked at a private dermatology practice in Singapore and complained of a crawling sensation on the skin and recurrent pruritic rashes when they were working in the storeroom. It was later discovered that there was water seepage in the wall caused by corrosion in a concealed copper pipe in the storeroom wall and black mould flourished on the damp wall and along joints between the bench-top and the wall. One of the nurses noticed numerous tiny, 1 mm grayish insects which appeared to jump. The nurses became convinced that these insects were the cause of their rashes and captured several of them in moist tissue paper placed overnight over the mould-infested areas. The clinic dermatologist noted 5–8 mm erythematous urticarial papules on the lower limbs of both nurses, especially around the knees and ankles (Figure 1). There were no excoriations or ulcerations and insects could not be found on the skin. The dermatologist prescribed 0.025% betamethasone valerate cream twice daily which quickly resolved the rash, without leaving any scars or marks. All their symptoms resolved when the seepage was rectified and the storeroom cleaned and repainted. Some of captured insects were given to the pest control personnel in charge of the medical centre and were confirmed to be Collembola. Tissue paper containing the Collembola was placed in ajar in a refrigerator at -4°C. Collembola were killed within minutes and were removed from the tissue and returned to the refrigerator for storage and extraction of protein. Some were thawed slowly at room temperature and preserved in absolute alcohol for further identification.

**Figure 2.**
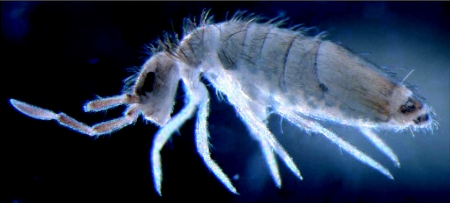
Collembola species of the genus *Drepanura* Schött (Collembola: Entomobryidae) caught along cracks in the damp mould infested store-room wall.

The Collembola were identified as a species of the genus, *Drepanura* Schött (Collembola: Entomobryidae) (Figure 2).

Skin prick tests to house dust and house dust mite were positive in one nurse and one volunteer. There was no prick test reaction to the other antigens in both the nurses and volunteer. Prick tests to the Collembola extract, skin crush tests and patch tests with whole Collembola were also negative in both the nurses and volunteers. IgE antibodies against Collembola proteins could not be detected in sera from the nurses. Full blood count, liver and renal function tests were normal in both nurses.

## Discussion

Collembola are entognathic, which is to say their mouthparts are within a buccal cavity. The chewing mouthparts, as well as the sucking mouthparts of some species are unlikely to be capable of biting (Pescott 1942). Rather, dermatitis or allergy has been attributed to the scales and cilia-like scales (chaetae) (Pescott 1942, Scott et al 1962, Mertens 2004).

The Collembola species caught by the nurses belonged to the genus, *Drepanura* Schött. This is interesting since this genus has not been previously reported from Southeast Asia, and the species is likely to be new to science. *Drepanura* has long ciliated chaetae on the body like many Entomobryidae and this led us to suspect, initially, that the urticarial papules in the nurses were due to an allergic (immunological) reaction or an urticating (histaminic) toxin present in the chaetae. However, the negative serum IgE tests exclude allergy and the negative skin prick tests with the Collembola extract and the skin crush tests with whole Collembola in both the nurses and the volunteers also argue against a Type 1 hypersensitivity reaction in the nurses and the presence of urticating chemicals in the Collembola. Negative patch tests also exclude the likelihood of a Type 4 delayed hypersensitivity reaction in the nurses.

There have been several cases reported of human skin reactions or crawling sensation in which Collembola have been found (Womersley 1939; Pescott 1942; Scott et al 1962; Amin 2001; Mertens 2004). In one clinical study of 20 patients with delusional parasitosis, 18 were found to have evidence of the presence of Collembola (Altschuler et al 2004). This evidence, however, was not based on the actual finding of Collembola in the skin scrapings but on ‘images of Collembola’ in digital pictures of skin scrapings. These images are disputable since they are the result of uncontrolled manual digital enhancement. As pointed out by Berenbaum (2005), this report may represent a case of pareidolia - ‘a type of illusion or misperception involving a vague or obscure stimulus being perceived as something clear and distinct’ (Carroll 2005). However, an allergic reaction to some species of Collembola may be possible. Mertens (2004) described a female patient who developed allergy from sitting on the cushion of a rattan chair. *Seira domestica* was found populating the hollow rattan branches and apparently left their hiding place at night to crawl all over the chair. The cushion reportedly collected many of the lost scales, causing the allergy. According to Mertens, the chair was on a warm and moist veranda. Such conditions are also conducive for infestation by moulds and other micro-organisms so the mere finding of Collembola does not necessarily establish a cause and effect relationship.

Janssens and Christiansen (2007) recently compiled all reported cases of Collembola affecting humans and concluded that little if any evidence existed of their allergic action. There has not been any attempt to test the ability of any Collembola species to cause human reactions using immunological methods as was done here. Based on our findings, it is unlikely that dermatitis, either through and allergic or direct histaminic action, was caused in the nurses and healthy volunteers by this undescribed *Drepanura* species.

One of the nurses had a positive prick test reaction to house dust mites. Urticarial lesions similar to those observed are known to occur in such individuals so it is possible that the lesions in that particular nurse are part of this phenomenon. Collembola generally inhabit moist environment, which may contain decaying organic matter, and organisms such as algae, fungi, bacteria, protozoa, mites and other arthropods. We believe that one or more of these might be the cause of skin reactions and suggest that future researchers should pay more attention to the other organisms and decaying organic material that also exist in the moist environment favoured by Collembola. Our report also highlights the shortcomings of self-collection of samples and the possible influence of the pareidolia phenomenon. Samples should, ideally, be collected by the investigator who should also pay attention to the ecosystem and its inhabitants.
